# Acute Tonsillitis

**Published:** 1884-02

**Authors:** F. Solis Cohen


### Selections.

---

### ACUTE TONSILLITIS—EFFICACY OF THE COMPOUND GUAIAC GARGLE AND OF THE SALICYLATE OF SODIUM.

For therapeutic purposes, according to the teaching of Prof. J. Solis-Cohen, and as practiced in his clinics, cases of acute tonsillitis may be divided into two classes: 1st, simple inflammatory or local; 2d, rheumatic or constitutional.

Each of these classes might be extensively subdivided, according to the most prominent objective or subjective, anatomical or pathological manifestations. Such refinement in nomenclature (for this alone is it, practically) is, however, unnecessary in arriving at the therapeutic indications. For whether the tonsillitis be unilateral or bilateral, or the two glands be affected consecutively—whether the inflammation be superficial or deep-seated, circumscribed or diffuse, limited to the follicles, or involving all the component structures—whether the morbid process be confined to the tonsil, or extend more or less widely among neighboring tissues—any particular case having been properly assigned to one of the two groups mentioned, its treatment need not be materially changed.

Of course, should suppuration threaten or occur, or should the enlargement of the tonsils be such that respiration becomes seriously impeded, incision, scarification, or other appropriate surgical procedure may be required. But when the patient is seen early, say within the first twenty-four hours, such accidents are very unlikely; if, indeed, the tendency to grave manifestations be not entirely averted. At all events, they did not arise in any of the cases treated at the Polyclinic.

The treatment has been as simple as it has been eminently successful; the principal reliance having been placed upon two remedial measures only, each of which seems entitled to be termed specifically appropriate for the particular group of cases in which it has been employed.

1. *Simple Inflammatory Tonsillitis.*—When the principal objective symptoms consist in alteration of voice, and more or less redness and enlargement of one tonsil, with or without swelling of the glands at the angle of the jaw; there being little constitutional disturbance; the pain complained of being chiefly due to tension; the odynphagia which is usually present having manifested itself after the swelling of the gland has been noticed; the disease may be considered as a local affection.

The treatment adopted in such instances is a modification of the old and well-tried guaiac treatment; and consists in the employment as a gargle of a mixture long known throughout the West and Southwest under the name of "diphtheria mixture" and similar titles. This is known to the House Pharmacopœia as the *Gargarysma Guaiaci Composita.* Two fluidrachms each, of the ammoniated tincture of guaiac and the compound tincture of cinchona, are mixed with six fluidrachms of clarified honey, and shaken together until the sides of the containing vessel are well greased. A solution, consisting of eighty grains of chlorate of potassium, in sufficient water to make four fluid-ounces, is then gradually added, the shaking being continued. If the apothecary is careful to make this preparation *secundum artem*, a not unpleasant mixture will be obtained. Without due care, however, the resin will be precipitated.

The patient is directed to gargle with this mixture freely and frequently, at intervals varying from every half-hour to every three hours. In some instances, a saline cathartic is first administered. Should any of the guaiac mixture be allowed, it is considered rather beneficial than otherwise, and its deglutition is sometimes recommended.

Relief is usually experienced within a few hours, and recovery is prompt. Patients often return only to report their cure.

A young colored man had been attacked about eight hours before presenting himself at the clinic. When the writer saw him, there was great enlargement of the left tonsil, causing intense pain, and preventing deglutition of solids. The submaxillary gland and some of the cervical glands of the same side were so much swollen that he was compelled to bend his head down toward the right shoulder. This patient reported himself well sixteen hours after coming under treatment. In his case, one ounce of Rochelle salts was first administered, and the compound guaiac gargle was directed to be used every two hours; but the relief following the first application was so grateful, that, in order to secure freedom from pain, the patient resorted to his medicine every half-hour or so.

A little girl of seven years was brought to the clinic with unilateral folliculous tonsillitis, a few hours after she had begun to complain of pain in her throat, and was told to use the compound guaiac gargle at intervals of two hours. She did not return until a month later, June 16, when she was brought for treatment of an acute coryza. Her mother remarked: "That medicine for her throat worked like a charm. She got worse for a few hours, but was well next morning."

Several similar cases of prompt relief could be cited in addition.

2. *Rheumatic or Constitutional Tonsillitis.*—When the first manifestation of the disease (excluding prodromata of headache, malaise, etc., which may or may not be present) is intense pain upon deglutition, causing great accumulation of saliva from unwillingness to swallow the excessive secretion; examination of fauces revealing perhaps a slight congestion—perhaps nothing more; more or less febrile reaction soon ensuing; the case may be assigned to the second or rheumatic group.

In these cases the odynphagia cannot be explained by anything visible upon inspection. It is sometimes referred to a point representing the entrance into the œsophagus, and the examination with the laryngoscopic mirror will show some redness of the mucous membrane covering the arytenoid and supra-arytenoid cartilages and the pharyngeal surface of the cricoid cartilages. More or less soreness in the throat is constantly present; respiration often becoming painful, and phonation excessively so.

Some hours after, as the headache, pulse, and temperature decline, one or both tonsils become enlarged; usually one consecutively to the other. The follicles are often distended with a caseous or sebaceous material, which, under the microscope, is seen to consist of scattered (pavement) epithelial cells, some oil globules, and a mass of granular detritus, mingled with which are the spores and filaments of the *Leptothrix buccalis*, *Oïdium albicans*,* rod-bacilli, and other fungi, as usually found in the secretions both of healthy and of diseased tonsils. Some drops of blood from an inflamed tonsil, in one of these cases, showed great deformity of the red corpuscles, and unusual size and number of the white corpuscles, which were more than ordinarily granular; but no micrococci could be detected therein. A drop of blood from the finger of the same patient presented nothing abnormal.

Usually, as the pain in the throat subsides, muscular soreness occurs in the neck, back, and loins, oftentimes in the sterno-cleido-mastoideus of the side corresponding with the tonsil first enlarging. Sometimes the soreness is experienced in one or more of the limbs; and some one of the larger joints may become more or less stiff, though neither red, swollen, nor painful Indeed, rheumatic or rheumatoid pains may flit from one portion of the body to another, during several days. In rare instances, transient albuminuria follows.

These cases are treated with salicylic acid or salicylate of sodium. The constipation usually present, due either to the disease or to the remedy, is relieved with an appropriate saline cathartic.

The following formula makes a pleasant and efficient mixture:

℞.—Sodii salicylatis..............................ʒij.
Ol. gaultheriæ..................................mj (vel. q. s.).
Liquoris ammonii citratis,
Syrupi simplicis............................*aa* f ℥ii.—M.

S.—A tablespoonful every two hours.

As soon as the pains are relieved, the intervals are lengthened; or sacillyate of quinine or of cinchonidine is substituted, as a

* Or something which I cannot distinguish from it. I have seen this fungus in but one case, and, curiously enough, in this instance there were no aphthæ upon the mucous membrane covering the hard palate; though I have observed aphthous sores in several of these cases, and especially in the neighborhood of the incisor teeth.

tonic, in five-grain doses, at intervals of four to six hours. Ringing in the ears (an occasional occurrence) calls for cessation of the sacillyates ; when it usually passes away. In one case, where persistent, it was relieved by small doses of the infusion of digitalis. During the progress of the more acute symptoms, the patient's comfort may be promoted by allowing small lumps of ice to melt in the mouth from time to time, or even by the use of the compound guaiac gargle.

Stiff-neck, the most annoying of the muscular complications, should it be severe, is relieved by faradization more promptly than by medication ; the negative electrode being applied to the painful spot, or moved along the course of the sterno-cleido-mastoid muscle, the positive electrode being grasped in the hand of the same side. The same measure is, of course, applicable to other muscles.—*F. Solis Cohen. Medical News.*

